# Emotion-induced loss aversion and striatal-amygdala coupling in low-anxious individuals

**DOI:** 10.1093/scan/nsv139

**Published:** 2015-11-19

**Authors:** Caroline J. Charpentier, Benedetto De Martino, Alena L. Sim, Tali Sharot, Jonathan P. Roiser

**Affiliations:** ^1^Institute of Cognitive Neuroscience, University College London, London WC1N 3AZ, UK,; ^2^Affective Brain Lab, Department of Experimental Psychology, University College London, London WC1H 0AP, UK, and; ^3^Department of Psychology, University of Cambridge, Cambridge CB2 3EB, UK

**Keywords:** decision-making, loss aversion, emotion, anxiety, functional magnetic resonance imaging (fMRI)

## Abstract

Adapting behavior to changes in the environment is a crucial ability for survival but such adaptation varies widely across individuals. Here, we asked how humans alter their economic decision-making in response to emotional cues, and whether this is related to trait anxiety. Developing an emotional decision-making task for functional magnetic resonance imaging, in which gambling decisions were preceded by emotional and non-emotional primes, we assessed emotional influences on loss aversion, the tendency to overweigh potential monetary losses relative to gains. Our behavioral results revealed that only low-anxious individuals exhibited increased loss aversion under emotional conditions. This emotional modulation of decision-making was accompanied by a corresponding emotion-elicited increase in amygdala-striatal functional connectivity, which correlated with the behavioral effect across participants. Consistent with prior reports of ‘neural loss aversion’, both amygdala and ventral striatum tracked losses more strongly than gains, and amygdala loss aversion signals were exaggerated by emotion, suggesting a potential role for this structure in integrating value and emotion cues. Increased loss aversion and striatal-amygdala coupling induced by emotional cues may reflect the engagement of adaptive harm-avoidance mechanisms in low-anxious individuals, possibly promoting resilience to psychopathology.

## Introduction

Detecting and processing changes in our environment and adapting our decisions in response to those changes, are important features of human behavior. For example, we are likely to behave and make choices differently if we receive positive or negative social feedback (Sip *et al*., 2015), or if threatening and emotionally arousing cues appear in our surroundings (Mobbs *et al*., 2007, 2009). However, such behavioral adaptation varies substantially across individuals, and the factors that influence how people alter their decision-making in emotional situations remain poorly understood.

Trait anxiety is likely to be an important factor in people’s tendency to alter their decisions in response to emotional cues. Rodent studies have reported that low levels of anxiety are associated with adaptive stress-coping and learning behavior (Landgraf and Wigger, 2002; Herrero *et al*., 2006). Highly anxious humans exhibit difficulty in modulating learning in volatile environments (Browning *et al*., 2015), cognitive control (Derryberry and Reed, 2002; Bishop, 2007, 2009) and emotion regulation (Etkin *et al*., 2010; Farmer and Kashdan, 2012); and it has been suggested that the flexible modulation of behavior in response to anxiogenic environmental changes may be an important mechanism by which further exposure to stress can be avoided (Mathews and Mackintosh, 1998; Robinson *et al*., 2013, 2015). Therefore it is possible that highly anxious individuals may fail to adapt their decision-making under emotional conditions. On the other hand, high anxiety is also associated with exaggerated responses to emotional stimuli (Etkin *et al*., 2004; Fox *et al*., 2007; Stein *et al*., 2007; Sehlmeyer *et al*., 2011), raising the possibility that decision-making in highly anxious individuals may be disproportionately influenced by emotion.

Therefore, we asked whether decision making is influenced by emotional cues to a greater extent in low-anxious individuals (potentially driven by greater behavioral flexibility) or in high-anxious individuals (potentially driven by greater emotional reactivity). To disambiguate between these hypotheses, we developed a functional magnetic resonance imaging (fMRI) paradigm where each decision (accepting or rejecting a gamble) was preceded by emotional or non-emotional primes. We examined people’s decisions in the framework of Prospect Theory (Kahneman and Tversky, 1979) and modeled their loss aversion [the tendency to overweigh potential losses relative to gains (Kahneman *et al*., 1991; Hardie *et al*., 1993)] under emotional relative to non-emotional conditions. To investigate whether avoidance of potential losses is altered specifically under threat, or under emotional arousal in general, we used both negative and positive emotional cues.

Based on prior work implicating the amygdala and ventral striatum in both loss aversion (Tom *et al*., 2007; De Martino *et al*., 2010; Canessa *et al*., 2013; Sokol-Hessner *et al*., 2013) and the processing of emotional cues (Adolphs, 2002; Glascher and Adolphs, 2003; Dalgleish, 2004; Mobbs *et al*., 2006; Phelps, 2006; Pessoa and Adolphs, 2010; Wang *et al*., 2014), we hypothesized that these regions would drive the influence of emotion on economic decisions. Specifically, we tested two mechanistic hypotheses: (i) that enhanced amygdala and striatum responses to potential losses relative to gains (‘neural loss aversion’) may be directly modulated by emotion in a manner that drives changes in behavior and (ii) that the amygdala and ventral striatum play complementary roles in this modulation of decision-making, and it is their functional integration (as opposed to their activation) that underlies changes in loss aversion.

## Materials and methods

### Participants

Thirty healthy volunteers were recruited by advertisement. Data from two participants were excluded because of a lack of behavioral consistency in the gambling task, making loss aversion impossible to model. Final analyses included 28 participants (15 males, 13 females, age range 19–47 years, mean 26.5 years). Participants gave written informed consent and were paid for their participation in an incentive-compatible manner. See Supplementary Methods for exclusion criteria and payment details. The study was approved by the local departmental ethics committee.

### Procedure

**Fig. 1.**
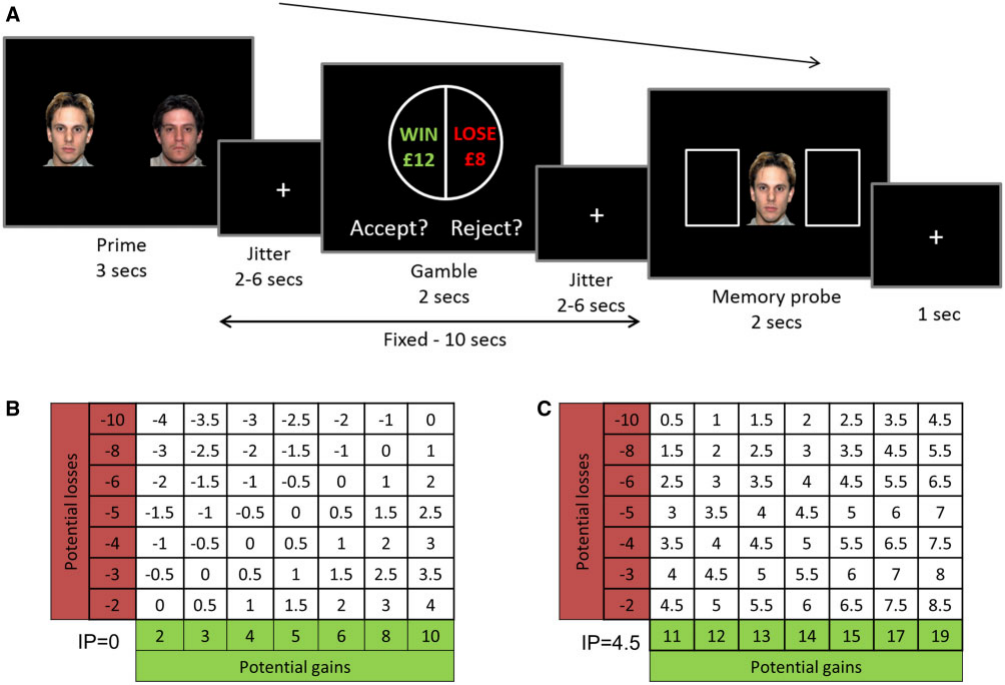
Experimental design. (A) On each trial, participants were first presented with an array (prime) of 2 or 4 faces (all happy, all fearful or all neutral) or objects (light bulbs) and had 3 s to memorize it. They then had to decide whether to accept or reject a mixed gamble in which there was a 50% chance of winning the amount in green, and a 50% chance of losing the amount in red. Finally, a probe from the first array was presented and participants had to report its position. (B, C) To optimize model fitting and sensitivity to emotional context, an estimate of each participant’s indifference point (IP) was obtained from the practice session and used to define the gamble gain/loss matrix. Each matrix was formed by combining seven potential gains with seven potential losses, leading to 49 gambles, repeated across each of the four conditions. Example matrices are shown with the resulting gamble expected value (EV = 0.5*gain + 0.5*loss), centered on an IP of 0 (B, non-loss averse participant) or 4.5 (C, highly loss averse participant).

Participants attended the laboratory on 2 different days. On Day 1 (screening session), participants were administered the Mini International Neuropsychiatric Interview (Sheehan *et al*., 1998), Beck depression inventory (BDI; Beck *et al*., 1961) and an MRI safety questionnaire. Eligibility for the study required: no past or present psychiatric disorders, including alcohol/substance dependence/abuse, BDI < 15 and no MRI contraindications. Participants were told that the aim of the study was to investigate how memory was affected by emotions. This cover story was used to avoid participants deducing the true goal of the experiment (demand characteristics: Orne, 2009), i.e. the manipulation of their gambling behavior by emotion. They first practiced the memory task (see Supplementary Methods for a full description). Then they were instructed that to make this memory task more challenging, they would perform a distracting gambling task while holding the stimuli in memory. They next completed a training block of gamble-only trials. Finally, they completed a training block of the combined emotional decision-making task (Figure 1A).

Participants returned to the laboratory for Day 2 (scanning session) after the screening session (mean delay = 17.92 days, range = 1–44 days). During this session, they initially completed one block of trials of the combined emotional decision-making task (Figure 1A) before entering the scanner, and four further 11-min blocks during fMRI scanning. Since there is large variability in loss aversion across individuals, each participant’s indifference point on the loss aversion task from Day 1 was used on Day 2 to individually tailor the gamble matrix (Figure 1B and C).

After the scan, participants completed the BDI a second time and the state-trait anxiety inventory (Spielberger *et al*., 1983). None of the 28 participants scored above 15 on the BDI at this time (mean = 1.68, s.d. ± 2.09, range 0–10). Mean trait anxiety was 31.2 (s.d. ± 6.34). Trait anxiety was used as a covariate in the analyses; in addition, a median split was performed, with 14 participants in a ‘low’ trait anxiety group (mean = 25.9, s.d. ± 3.03, range 20–30) and 14 in a ‘high’ trait anxiety group (mean = 36.5, s.d. ± 3.65, range 33–44).

### Emotional decision-making task

Each trial started with the presentation of either two or four prime stimuli from the same condition (happy/fearful/neutral/object) for 3 s (prime: Figure 1A). Participants were instructed to memorize their location. After a jittered delay of 2–6 s, the gamble appeared for 2 s and participants decided whether to accept or reject this gamble. There was another 2–6 s jittered delay before the probe face/object appeared. Participants had 2 s to indicate the location where the probe had been displayed in the first screen, followed by a 1 s fixation cross between trials. Gamble outcomes were not revealed. The two delays were jittered to decorrelate the prime stimuli from the gamble presentation time, but always summed to 8 s, such that the intertrial interval was maintained at a constant 16 s throughout the task. Participants completed 196 trials of this combined task (49 trials of each of the four conditions: happy, fearful, neutral, object). Gambles were randomly sampled from a 7*7 gain–loss matrix centered on each participant’s own indifference point (example matrices: Figure 1B and C). This was done to ensure that the same ranges of wins and losses were presented for each emotion condition, and to optimize sensitivity to detect emotion-driven changes in loss aversion with a majority of gambles close to the participant’s indifference point.

### Behavioral data analysis

Behavioral data were analyzed using IBM SPSS Statistics (v.21) and Matlab. Missed gamble trials were excluded. For each participant, the probability of accepting the gamble (P_accept_), mean reaction time (RT) to accept or reject the gamble, number of missed trials and working memory accuracy were calculated separately for the different emotion conditions and submitted to repeated-measures analysis of variance (ANOVA) to assess the impact of emotion on behavior. Trait anxiety scores were added as covariates in the analyses (Supplementary Table S1).

To assess loss aversion a two-parameter model was estimated based on Prospect Theory’s subjective utility function using a maximum likelihood estimation procedure in Matlab (Kahneman and Tversky, 1979; Sokol-Hessner *et al*., 2009; Chib *et al*., 2012). For each trial, the subjective utility (*u*) of each gamble was estimated using the following equation (with losses coded as negative values):

(1) u(gamble)=0.5*gain+0.5*λ*loss

where λ is the ‘loss aversion’ parameter: λ > 1 indicates an overweighing of gains relative to losses and λ < 1 the converse. These subjective utility values were used in a softmax function to estimate the probability of accepting each gamble (coded as 0 or 1 for each rejected or accepted gamble, respectively):

(2) $$P\left(\text{gamble acceptance}\right)={\left(\text{1}+\text{exp}(-µ*u\left(\text{gamble}\right))\right)}^{-}{}^{\text{1}}$$

where µ is the logit sensitivity or ‘inverse temperature’ parameter, an index of choice consistency for repeated identical gambles, equivalent to the maximal slope of a logistic regression curve: higher µ values indicate more consistent choices.

Different models were run using this procedure, where loss aversion (λ) and choice consistency (µ) parameters were estimated: (i) across all trials; (ii) separately for emotional (happy and fearful faces) and non-emotional (neutral faces and objects) trials or (iii) separately for each of the four emotion conditions. Model comparison analyses using Bayesian Information Criteron (BIC) (Schwartz, 1978) revealed that the two-condition model was more parsimonious than the four-condition model (see Supplementary Methods for details). Therefore, the two-condition model was used preferentially in all analyses, except to verify that the effects obtained were independent of valence or specific to emotion rather than faces in general. The percentage change in λ and in μ between emotional and non-emotional conditions was calculated from this two-condition model. Both variables were normally distributed with Skewness values smaller than 1 and Kurtosis values smaller than 3 (percentage change in λ: Skewness = −0.252, Kurtosis = 0.669; percentage change in µ: Skewness = 0.672, Kurtosis = 2.033). However, the distribution of the loss aversion parameter λ was positively skewed, so when analyses where run on this parameter *per se* (e.g. correlation between loss aversion and trait anxiety), λ values were log-transformed before running statistical tests.

To estimate risk aversion we used a procedure reported previously (De Martino *et al*., 2010), based on the behavioral sensitivity to gamble variance (O’Neill and Schultz, 2010). When gamble variance is high (e.g. win £10/lose £10 relative to win £2/lose £2), the risk is high; therefore, risk averse individuals will exhibit a stronger reduction in gamble acceptance as gamble variance increases. To calculate this sensitivity to gamble variance, a linear regression was run between gamble variance [calculated for each gamble as (0.5*gain − 0.5*loss)^2^] and the probability of gamble acceptance (calculated for groups of gambles with the same variance). For each subject, risk aversion was approximated by the negative value of this regression slope, separately for emotional and non-emotional conditions. Note that the design of the task did not allow us to concurrently estimate loss and risk aversion in the same utility model. To do so, the task should have included a subset of trials where risk is present, but losses do not need to be weighed against gains, so that the model can distinguish between risk and loss aversion. However, because of fMRI time constraints, we were not able to add these trials to the task. Risk aversion was therefore estimated separately and added as a covariate in the analyses to ensure it did not affect the results. In particular, to ensure that the influence of trait anxiety was specific to the change in loss aversion, partial correlations were conducted, where emotion-driven change in loss aversion was correlated with trait anxiety while controlling for changes in risk aversion and choice consistency.

### MRI data acquisition and analysis

#### Acquisition parameters

Neuroimaging data were collected on a Siemens Avanto 1.5 T MRI scanner using a 32-channel head coil. To correct for inhomogeneities of the static magnetic field, fieldmaps were acquired and used in the unwarping stage of data preprocessing. Four functional scanning sessions, composed of 4 dummy and 203 functional volumes, were acquired using a pre-scan normalized gradient echo-planar imaging sequence with the following parameters: volume repetition time = 3.132 s, echo time = 50 ms, flip angle = 90°, matrix = 64 × 64, voxel size = 3 × 3 × 3 mm^3^, 36 axial slices sampled for whole-brain coverage, tilt = −30°. A T1-weighted magnetization-prepared rapid gradient-echo (MP-RAGE) anatomical scan was acquired at the end of the session (176 sagittal slices, repetition time = 2.73 s, echo time = 3.57 ms, flip angle = 7°, matrix = 224 × 256, voxel size = 1 × 1 × 1 mm^3^).

#### Statistical analyses

MRI data preprocessing and analysis were performed using Statistical Parametric Mapping (SPM8) software (Wellcome Trust Centre for Neuroimaging, London, UK, http://www.fil.ion.ucl.ac.uk/spm) in Matlab. Preprocessing included field map correction, realignment, unwarping, coregistration, spatial normalization and smoothing (see Supplementary Materials for details). For each participant, the general linear model was used to model blood oxygen level-dependent (BOLD) signals during the task, incorporating an autoregressive [AR(1)] model of serial correlations and a high-pass filter at 1/128 s.

Two first-level models were defined. The first model identified brain regions tracking gain and loss value independent of emotion (similar to Tom *et al*., 2007). It included the following regressors (and associated durations), collapsed across all memory conditions and convolved with the SPM synthetic hemodynamic response function: prime onset (3 s); gamble onset (2 s) with gain value, loss value (coded as negative values) and choice difficulty (distance between gamble expected value and participant’s indifference point) as parametric modulators; memory probe onset (stick function); missed gamble onset (if any: stick function). The six movement parameters were also included in the model.

To assess whether these responses were modulated by emotion, another model contained the same regressors as above but separately for emotional trials (happy and fearful faces) and non-emotional trials (neutral faces and objects). This constituted our primary analysis based on the behavioral results suggesting that grouping trials into emotional and non-emotional ones was most parsimonious. However, to ensure that our effects were not driven by face processing *per se* and to contrast emotional with neutral faces, we estimated a further model in which neutral face and object trials were modeled separately.

First-level contrasts were created through linear combinations of the resulting beta images and analyzed at the group level with one-sample *t*-tests, using the standard summary-statistics approach to random-effects analysis in SPM. A cluster-forming threshold of *P* < 0.001 uncorrected was applied, followed by family-wise error (FWE) correction at *P* < 0.05, using small-volume correction (SVC) in our a priori regions of interest (ROIs). These were bilateral ventral striatum (caudate and putamen, left and right combined) given its role in loss aversion (Tom *et al*., 2007; Canessa *et al*., 2013) and bilateral amygdala, given its role in processing emotion (Adolphs, 2002; Glascher and Adolphs, 2003; Dalgleish, 2004; Phelps, 2006; Pessoa and Adolphs, 2010; Wang *et al*., 2014). ROIs were anatomically defined using the automated anatomical labeling atlas in the SPM WfuPickAtlas toolbox (Tzourio-Mazoyer *et al*., 2002).

#### Functional connectivity analyses

Amygdala-striatum functional connectivity was analyzed using psychophysiological interaction (PPI) in SPM8. First, the ventral striatum cluster found to track value (using a *P* < 0.001 (uncorrected) threshold and masked with the anatomical ROI) was used as the seed region. BOLD time series was extracted across all voxels in this mask and adjusted for all effects of interest. A first-level model was created for each participant including the deconvolved striatal BOLD timeseries (physiological regressor), the emotional context (contrast between emotional and non-emotional trials at the time of the gamble: psychological regressor) and their cross-product (PPI regressor). This model also included the following regressors: prime onset, memory probe onset, parametric modulators for gains and losses at gamble onset, all split by emotion condition, as well as missed gamble onset (if any) and movement regressors. Two nuisance timeseries were also added, from a white-matter voxel (corpus callosum body, Montreal Neurological Institute (MNI) coordinates: 0,14,19) and from a cerebrospinal fluid voxel (center of right lateral ventricle, MNI coordinates: 4,14,18); both in the same *y*-plane as the ventral striatum peak voxel. Finally, contrasts were defined on the striatal timeseries (physiological) regressor, modeling ‘main effect’ functional connectivity and on the PPI regressor, modeling the modulation of functional connectivity by emotional context, which were analyzed at the second level. Again, to make sure that our effects were driven by emotional faces rather than faces in general, the PPI analysis was repeated for emotional *vs* neutral faces trials at the time of the gamble (excluding the object condition).

## Results

### Emotional modulation of loss aversion depends on trait anxiety

**Fig. 2.**
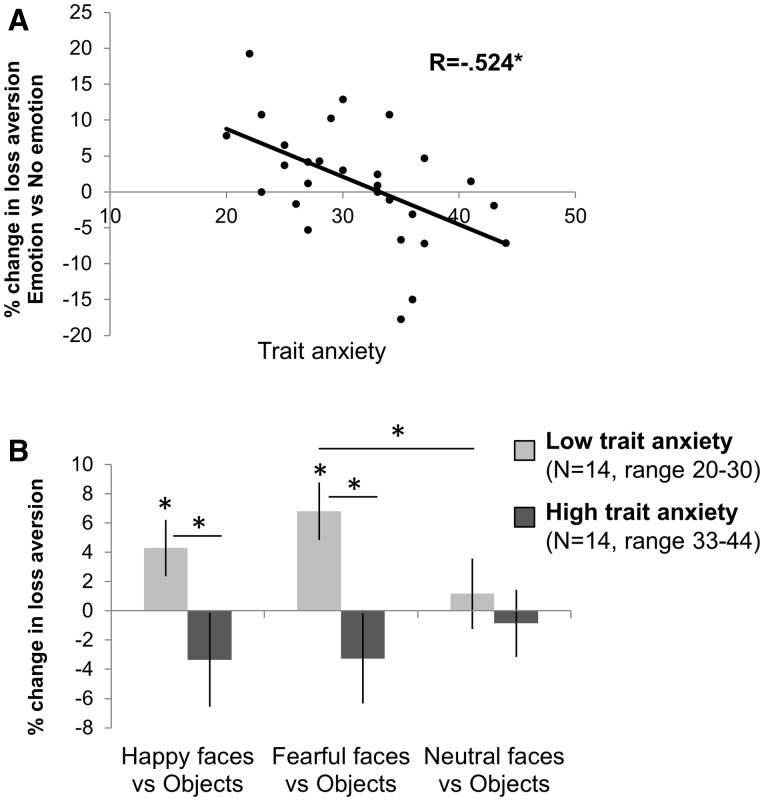
Emotional cues modulate loss aversion in low-anxious individuals. (A) The change in loss aversion following emotional relative to non-emotional primes was negatively correlated with trait anxiety across participants. (B) Participants with low trait anxiety (median-split, *N* = 14 per group) showed a significant increase in loss aversion following both happy and fearful stimuli. Collapsing fearful and happy trials into an emotional condition and neutral and object trials into a non-emotional condition was justified by the fact that there was no valence effect, and no differences between neutral faces relative to object stimuli. Two-tailed *P*-values: *P < 0.05. Error bars represent SEM.

Emotional stimuli increased loss aversion (λ) in individuals with low trait anxiety. The percentage change in loss aversion was calculated for each participant between emotional and non-emotional trials, thus removing interindividual variability due to loss aversion values *per se*, and related to trait anxiety scores across participants. We identified a significant negative relationship between emotion-driven change in loss aversion and trait anxiety (*r*_(28)_ = − 0.524, *P* = 0.004, Figure 2A), such that low-anxious individuals showed the greatest increase in loss aversion induced by emotional cues. Importantly, baseline loss aversion (modeled across all trials independent of emotion condition and log-transformed because positively skewed) was not correlated with trait anxiety (*r*_(28)_ = −0.031, *P* = 0.88; after removing one outlier with very high λ: *r*_(28)_ = 0.043, *P* = 0.83), suggesting that the effect of trait anxiety on loss aversion change is not simply driven by regression to the mean.

The percentage change in risk aversion between emotional and non-emotional trials, although highly correlated with change in loss aversion (*r*_(28)_ = 0.65, *P* < 0.001; expected given that loss and risk aversion were estimated separately; see De Martino *et al*., 2010; Canessa *et al*., 2013), was not correlated with trait anxiety (*r*_(28)_ = −0.23, *P* = 0.24). Finally, the correlation between emotion-induced change in loss aversion and trait anxiety was unchanged when controlling for change in risk aversion and change in choice consistency (partial correlation, *r*_(28)_ = −0.51, *P* = 0.008).

Performing a median split on trait anxiety scores confirmed the emotional modulation of loss aversion, revealing a significant condition (emotion/no emotion) * trait anxiety (low/high) interaction (*F*_(1,26)_ = 6.96, *P* = 0.014). Although the ‘low’ anxious group showed a significant increase in loss aversion following emotional relative to non-emotional stimuli ( + 5.49%, s.d. = 6.38%, *t*_(13)_ = 3.22, *P* = 0.007), there was no significant effect of emotion on loss aversion in the ‘high’ trait anxiety group (−2.81%, s.d. = 7.52%, *t*_(13)_ = −1.40, *P* = 0.18).

To check that collapsing emotional (happy/fearful) and non-emotional (neutral/object) conditions together did not alter the results, we examined the change in loss aversion separately for each condition (happy, fearful and neutral) relative to object (Figure 2B). First, there was no difference between positive and negative emotion: the valence (happy/fearful) * trait anxiety group (low/high) interaction was non-significant (*F*_(1,26)_ = 0.24, *P* = 0.63) and the effect of trait anxiety group on emotionally driven changes in loss aversion was significant for happy and fearful faces separately (happy: *t*_(26)_ = 2.04, *P* = 0.05; fearful: *t*_(26)_ = 2.75, *P* = 0.01). Second, the effect of neutral faces relative to object stimuli on loss aversion did not differ between trait anxiety groups (*t*_(26)_ = 0.6, *P* = 0.55). Third, model comparison analyses (see Supplementary Methods) showed that estimating loss aversion for emotional and non-emotional trials (i.e. collapsing happy and fearful together, and neutral and object together) was more parsimonious than estimating loss aversion separately for all conditions.

Analyses of the choice consistency parameter (μ) revealed that participants were more consistent in their gambling choices on emotional than non-emotional trials, though this effect did not correlate significantly with trait anxiety (see Supplementary Results and Figure S1). Similarly, only the emotional modulation of gamble acceptance and loss aversion correlated significantly with trait anxiety, ruling out the possibility that differences in memory, RTs or missed trials may have driven the observed effects (Supplementary Table S1).

Taken together, our behavioral results suggest that emotional cues trigger changes in loss aversion as a function of trait anxiety, such that low-anxious individuals show the greatest emotionally-induced increase in loss aversion. In addition, we reveal that this effect is not driven by risk aversion or by choice consistency. Finally, and surprisingly, both positive and negative emotional stimuli have a similar effect.

### Neural responses to decreasing losses are greater than to increasing gains

The first step in our fMRI data analysis was to verify that expected value signals were observed in the brain at the time of gamble, with exaggerated responses to losses (‘neural loss aversion’) in the ventral striatum. Next, we examined responses to emotional primes as well as emotional modulation of value signals in the amygdala. Finally, we investigated the interaction between these two regions using functional connectivity. For all analyses, we additionally assessed the relationship with trait anxiety.

**Fig. 3.**
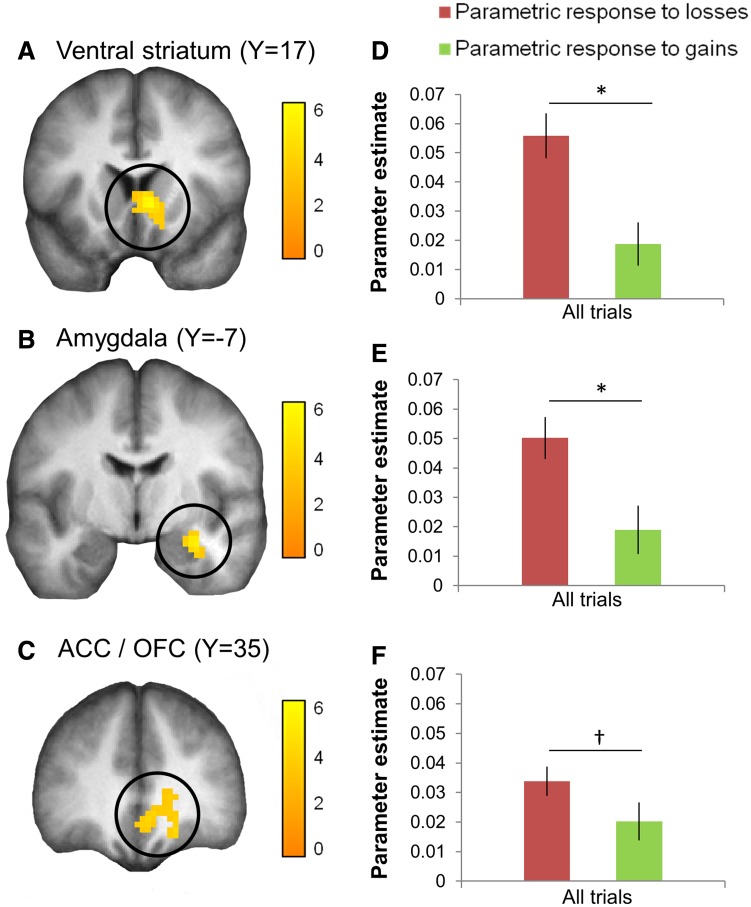
Brain regions tracking gamble expected values. A whole-brain analysis was conducted to identify regions showing a parametric response to decreasing losses and to increasing gains. Clusters surviving whole-brain FWE correction were found in the ventral striatum (A), the amygdala extending into the hippocampus (B) and the ACC/OFC (C). Activations are displayed at *P* < 0.001 (uncorrected) on the average anatomical scan from all 28 participants. Color bars represent *T*-values. (D–F) Parameter estimates (betas) extracted from the parametric response to losses (red bars) and to gains (green bars) separately revealed greater tracking of losses relative to gains in these regions (at trend level in the ACC). Note that the latter contrasts are orthogonal to that used for voxel identification and therefore do not require correction for a voxel-wise search. Two-tailed *P*-values: *P < 0.05, †*P* < 0.1. Error bars represent SEM.

A whole-brain analysis was first conducted to identify clusters with a parametric response to decreasing losses and increasing gains, time-locked to the presentation of the decision and independent of emotion condition. This gain and loss contrast is equivalent to a single parametric modulator representing the expected value of the gamble (0.5*gain + 0.5*loss, with losses coded as negative values). Three clusters surviving whole-brain correction for multiple comparisons were found to track gamble value (Supplementary Table S2A), located in the right ventral striatum (Figure 3A), right amygdala/hippocampus (Figure 3B) and anterior cingulate/orbitofrontal cortex (ACC/OFC) (Figure 3C), confirming previous reports of generic value signals in these regions (Gottfried *et al*., 2003; Tom *et al*., 2007; Kable and Glimcher, 2009; Morrison and Salzman, 2010). Parameter estimates (betas) were extracted for each region, separately for losses and gains. We identified a greater parametric response to decreasing losses relative to increasing gains in each of these three regions (significant in the ventral striatum, *t*_(27)_ = 3.52, *P* = 0.002; and amygdala, *t*_(27)_ = 3.16, *P* = 0.004; marginally significant in the ACC/OFC, *t*_(27)_ = 1.95, *P* = 0.06; Figure 3D–F), consistent with previous reports of loss-biased value signals in these regions (Tom *et al*., 2007; Canessa *et al*., 2013; Sokol-Hessner *et al*., 2013). We confirmed the result in the ventral striatum by creating a loss minus gain contrast (similar to Tom *et al*., 2007). This voxel-wise search yielded a cluster in the ventral striatum that responded more strongly to decreasing losses than increasing gains, overlapping with that reported earlier. This response correlated with trait anxiety across individuals, a result presented and discussed in Supplementary Results.

### Amygdala response to emotional cues correlates positively with trait anxiety

**Fig. 4.**
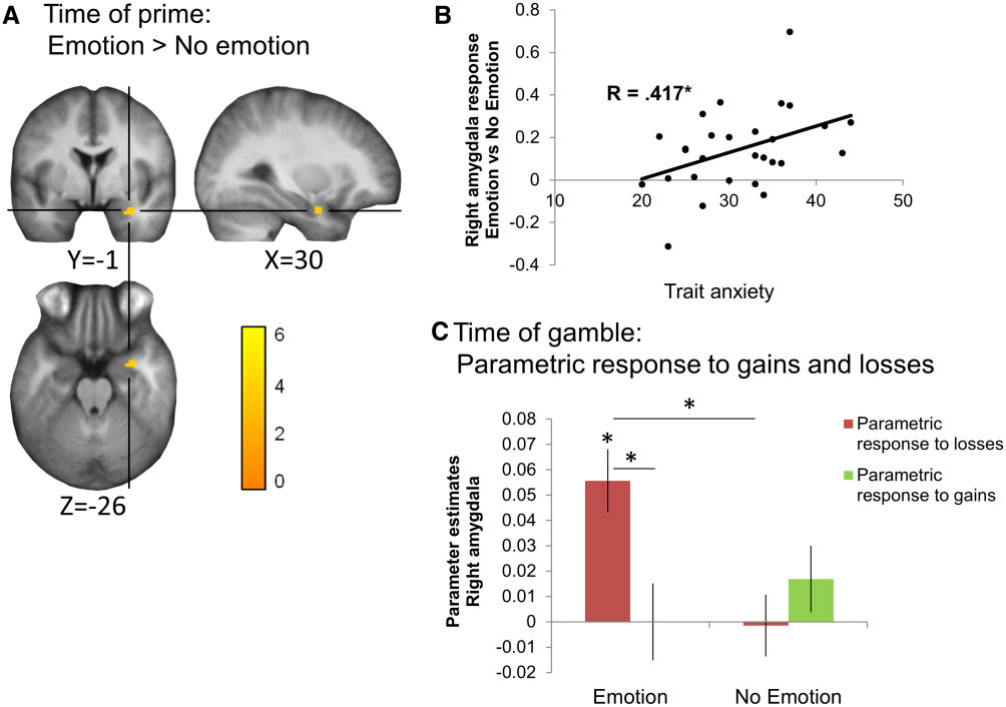
Modulation of amygdala responses by emotional cues. (A) A cluster in the right amygdala showed greater response to emotional *vs* non-emotional primes (i.e. at initial stimulus presentation). Activation is displayed at *P* < 0.001 (uncorrected), but survived FWE voxel-level SVC (*P*_SVC_ < 0.05) in the anatomically defined bilateral amygdala ROI. The color bar represents *T*-values and voxels are overlaid on the average anatomical scan from all 28 participants. (B) Amygdala response to emotional primes was positively correlated with trait anxiety. (C) Extracting parametric response to losses and to gains in this amygdala cluster at the time of gamble, separately for emotion and no emotion trials, revealed that the amygdala only tracks decreasing losses following emotional cues. Two-tailed *P*-values: *P < 0.05. Error bars represent SEM.

In a second analysis, time-locked to the presentation of the prime, we contrasted emotion (happy and fearful faces) with non-emotion (neutral faces and objects) trials. Consistent with prior reports (Sabatinelli *et al*., 2011), this revealed a widespread pattern of activation, with whole-brain corrected results in the fusiform gyrus, occipital gyrus, OFC/medial prefrontal cortex, posterior cingulate cortex, middle temporal gyrus, anterior insula, inferior temporal gyrus extending into frontal gyrus and amygdala extending into bilateral hippocampus (Supplementary Table S3). Given previous literature suggesting that amygdala responses vary with anxiety (Etkin *et al*., 2004; Stein *et al*., 2007; Sehlmeyer *et al*., 2011), we extracted signal from the right amygdala cluster (peak voxel MNI coordinates: 33,−1,−26; Figure 4A) and identified a significant positive relationship with trait anxiety (*r*_(28)_ = 0.417, *P* = 0.027; Figure 4B). This relationship was not driven by responses to faces in general: while amygdala responses to emotional relative to neutral faces correlated with trait anxiety (*r*_(28)_ = 0.39, *P* = 0.04), amygdala responses to neutral faces relative to objects did not (*r*_(28)_ = −0.042, *P* = 0.83; marginally significant difference between the two correlations: Steiger’s *Z* = 1.83, *P* = 0.067).

### Emotional cues modulate loss aversion signals in the amygdala

To test whether the earlier amygdala responses play a role in the observed emotion-driven changes in loss aversion, parametric responses to losses and to gains (at the time of the gamble) were extracted separately for emotional and non-emotional trials, from the right amygdala cluster identified earlier (responding to emotional primes—MNI: 33,−1,−26). The resulting parameter estimates (betas) were submitted to a 2 − (gamble component: loss/gain) by −2 (prime: emotional/non-emotional) repeated-measures ANOVA. There was a significant interaction (*F*_(1,27)_ = 7.998, *P* = 0.009, Figure 4C), driven by a positive amygdala parametric modulation, on emotional trials only, by decreasing losses (*t*_(27)_ = 3.61, *P* = 0.001) but not increasing gains (*t*_(27)_ = −0.88, *P* = 0.39). However, there were no relationships with trait anxiety or emotion-elicited change in loss aversion (added as covariates: all *P* > 0.25).

The earlier modulation of amygdala value signal was specific to emotional cues, rather than faces in general. When extracting the response to losses separately for emotion, neutral and object conditions, and submitting the resulting betas to a one-way ANOVA (emotion/neutral/object), there was a significant main effect of emotion (*F*_(2,54)_ = 3.49, *P* = 0.038). The amygdala parametric response to losses was higher on emotional face relative to neutral face trials (*t*_(27)_ = 3.08, *P* = 0.005) but not on neutral face relative to object trials (*t*_(27)_ = −0.6, *P* = 0.55).

### Striatal-amygdala functional connectivity is associated with changes in loss aversion

**Fig. 5.**
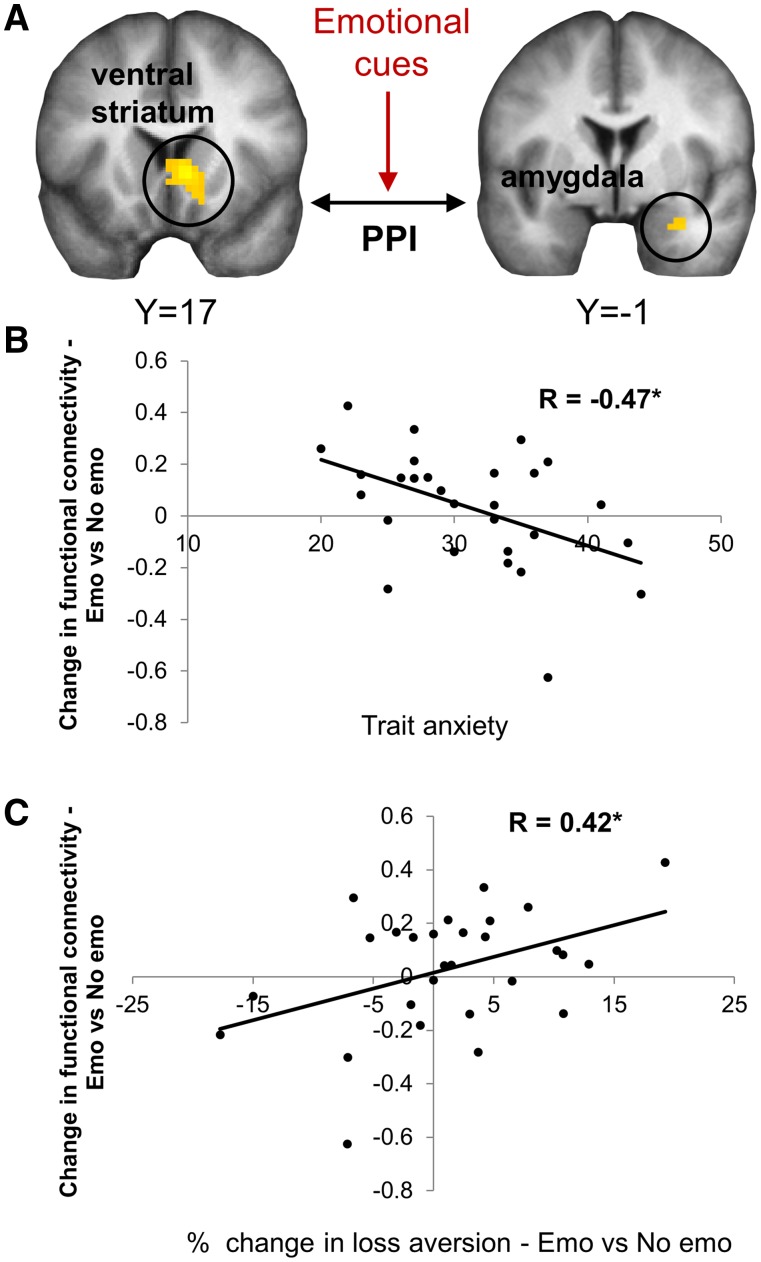
Emotional modulation of striatum-amygdala functional connectivity is related to trait anxiety and loss aversion change. (A) PPI analysis was conducted to assess how ventral striatum-amygdala functional connectivity was modulated by emotional relative to non-emotional cues, using the ventral striatum cluster as a seed (Figure 3A). (B) The PPI effect (i.e. emotion-driven increased connectivity) in the amygdala was negatively correlated with trait anxiety. In other words, low-anxious individuals exhibited increased ventral striatum-amygdala connectivity following emotional relative to non-emotional stimuli. (C) This increased functional connectivity was associated with emotion-elicited increase in loss aversion across participants.

Are emotionally induced changes in loss aversion driven by ventral striatum-amygdala interactions during emotional decision-making? To test this hypothesis, we conducted a PPI, with emotion *vs* non-emotion (at the time of the decision) as the psychological factor (Figure 5A). The ventral striatum cluster (shown in Figure 3A) was defined as the seed region, and beta estimates for the physiological (see Supplementary Results) and PPI effects were extracted from the right amygdala cluster that responded to emotional cues (the target region: shown in Figure 4A). The increase in ventral striatum-amygdala connectivity between non-emotional and emotional trials was negatively correlated with trait anxiety (*r*_(28)_ = −0.47, *P* = 0.012, Figure 5B), and positively correlated with emotion-elicited change in loss aversion (*r*_(28)_ = 0.42, *P* = 0.025, Figure 5C). In other words, low-anxious individuals exhibited an increase in striatal-amygdala functional connectivity on emotional trials, which in turn was associated with emotion-elicited loss aversion.

Again, this pattern of results held when examining the emotion-driven change in striatal-amygdala connectivity, excluding the object condition (negative correlation between PPI and trait anxiety: *r*_(28)_ = −0.38, *P* = 0.047), suggesting that trait anxiety modulates functional connectivity changes specifically in response to emotional stimuli.

## Discussion

How people alter their decisions in response to emotional cues, and the neural mechanisms underlying such changes, vary with their level of trait anxiety. Specifically, we reveal that low-anxious individuals exhibit increased loss aversion when primed with emotional cues. This was accompanied by and associated with increased functional coupling between the striatum and amygdala, regions that have been implicated in loss aversion (Tom *et al*., 2007; De Martino *et al*., 2010; Canessa *et al*., 2013; Sokol-Hessner *et al*., 2013).

One of our main aims was to establish whether loss aversion would be modulated by emotional cues to a greater extent in low-anxious individuals (which would be predicted by greater behavioral flexibility) or in high-anxious individuals (which would be predicted by emotional hypersensitivity). Our data support the first hypothesis. This is consistent with a recent study in which only low-anxious individuals decreased risk-taking under stress (Robinson *et al*., 2015). We suggest that this finding may reflect an adaptive ability of individuals with low anxiety to deploy harm-avoidance strategies (avoiding potential harm from monetary losses) in response to emotionally arousing cues. This could be linked with the reduced sensitivity to pathological anxiety disorders in this low-anxiety group (Robinson *et al*., 2013, 2015) and with previous reports of anxiety-related impairments in the ability to adapt behavior to changes in the environment (Blanchette and Richards, 2003; Farmer and Kashdan, 2012; Robinson *et al*., 2013, 2015; Browning *et al*., 2015).

An alternative interpretation of our findings could be that high-anxious individuals may in fact exhibit greater attentional control than low-anxious individuals and be better at ignoring the emotional primes, which are irrelevant to the gambling task. Although this interpretation is inconsistent with the theory of impaired attentional control in anxiety (Eysenck *et al*., 2007; Bishop, 2009), it remains possible that such superior attentional control is a feature of non-clinical anxiety (i.e. high trait anxiety in healthy individuals; see Robinson *et al*., 2013 for a review), and that dysfunctional attentional control only emerges in clinical anxiety. Further work is needed to distinguish between these explanations.

Recent literature has shown a growing interest in the link between anxiety and decision-making (for a review see Hartley and Phelps, 2012) and provided evidence for heightened sensitivity to uncertainty and ambiguity in high anxiety. A recent study demonstrated an increased framing effect in high-anxious individuals (Xu *et al*., 2013). According to Prospect Theory (Kahneman and Tversky, 1979), framing effects on choice could be driven both by loss aversion and by diminishing sensitivity to changes in value as value increases (resulting in risk avoidance in the gain domain and risk seeking in the loss domain). In our data, we did not find a direct relationship between trait anxiety and loss aversion, suggesting that the increased framing effect observed in Xu *et al*. (2013) may be driven by stronger diminishing sensitivity to value changes in high trait anxious individuals, rather than by increased loss aversion. Similarly, our data is in line with a recent study in adolescents showing that clinically anxious and healthy adolescents did not differ in their level of loss aversion (Ernst *et al*., 2014). We note that our sample of healthy volunteers included a relatively constrained range of anxiety scores; it would therefore be interesting to examine loss aversion (and its modulation by emotion) in clinically anxious individuals.

Our findings revealed that positive and negative emotional expressions induced similar changes in decision-making. This supports the hypothesis that increased avoidance of potential losses is recruited under general emotional arousal, rather than specifically under incidental threat. Recent work, using a pharmacological manipulation of autonomic arousal, suggests that arousal responses specifically drive loss aversion, but not risk aversion (Sokol-Hessner *et al*., 2015). Although speculative, this hypothesis of an arousal-driven loss aversion could explain our findings that (i) manipulating emotional arousal influenced loss aversion in the same direction regardless of valence and (ii) this effect was specific to loss aversion, with risk aversion (estimated separately) not altered by emotional manipulation.

However, due to time constraints in the scanner, a limitation of our task design was that we were not able to include additional trials necessary to estimate risk aversion together with loss aversion in the same utility model. Typically this would be done by adding choices between a sure gain and a gamble (featuring a chance of a higher gain or zero); on such gain-only trials only risk aversion (but not loss aversion) should contribute to safe choices. Without these trials, our Prospect Theory-derived model could not distinguish between risk and loss aversion. Estimating risk aversion separately, using an approach that has been used before (De Martino *et al*., 2010; Canessa *et al*., 2013), and ensuring it did not contribute to the results by adding it as a covariate in our analyses was the best alternative to overcome this limitation.

Our fMRI results shed light on a potential mechanism underlying the emotional modulation of economic behavior, related to amygdala-striatum functional connectivity. Consistent with previous studies, we found that both amygdala and ventral striatum tracked losses more strongly than gains (Tom *et al*., 2007; De Martino *et al*., 2010; Canessa *et al*., 2013; Sokol-Hessner *et al*., 2013); however, the modulation of these signals by emotional cues was not associated with emotionally-driven changes in loss aversion.

Instead, we found that the interaction between amygdala and ventral striatum was the neural metric most related to the behavioral effects we observed. Emotionally-induced changes in functional connectivity between ventral striatum and amygdala correlated negatively with trait anxiety and were associated with behavioral changes in loss aversion, with low-anxious individuals showing increased loss aversion together with increased amygdala-striatum functional connectivity in response to emotional cues. We note a potential concern that amygdala activations, according to a recent study, could be driven by drainage from nearby vessels such as the basal vein of Rosenthal (BVR: Boubela *et al*., 2015). However, in our fMRI data we did not observe patterns consistent with BVR signals, even at a very liberal threshold (data not shown). In particular, our amygdala cluster responding to emotional relative to non-emotional primes (MNI coordinates [30,−1,−24]) did not extend to the posterior amygdala, and instead was located in the lateral anterior amygdala on the opposite side to the BVR (MNI coordinates [14.6,−7.7,−15.5] according to Boubela *et al*., 2015) with voxels adjacent to the BVR not activated. Therefore, we believe that our amygdala activations are unlikely to be confounded by a contribution from the BVR.

Amygdala-striatum connectivity is well established in both animal and human fMRI work and has been suggested to play a role in motivated behavior (Price, 2003; Zorrilla and Koob, 2013), emotional memory (Ferreira *et al*., 2008; Paz and Pare, 2013), reward-related processes (Everitt and Robbins, 1992; Camara *et al*., 2008) and learning to avoid harmful negative outcomes (Delgado *et al*., 2009). Our results provide a further insight into a potential function of amygdala-striatum interactions, suggesting that changes in functional connectivity between these two regions, as opposed to responses in each region *per se*, may drive the tendency toward more conservative decisions under emotionally arousing conditions.

In summary, we show that incidental emotional cues can modulate loss averse behavior and associated neural responses, shedding light on a potential mechanistic account of emotional influences on economic decisions (Phelps *et al*., 2014). We speculate that the amygdala may integrate emotional information about external cues together with value information from the ventral striatum, to produce a decision signal. Individual differences in amygdala-striatal coupling are related to trait anxiety, possibly reflecting improved functional integration between these regions in low-anxious individuals and greater flexibility to adapt decision-making in emotionally volatile environments.

## Supplementary Material

Supplementary Data
