# Supplementary material for: Emotion-induced loss aversion and striatal-amygdala coupling in low-anxious individuals
Source: Soc Cogn Affect Neurosci. 2015 Nov 19;11(4):569–79. doi: 10.1093/scan/nsv139 (PMC4814785; doi:10.1093/scan/nsv139)
Supplement: Supplementary Data [file supp_11_4_569__index.html]

Emotion-induced loss aversion and striatal-amygdala coupling in low-anxious individuals — Emotion-induced loss aversion and striatal-amygdala coupling in low-anxious individuals — Supplementary Data 

# Emotion-induced loss aversion and striatal-amygdala coupling in low-anxious individuals

## Supplementary Data

files

- Supplementary Data - doc file
