# Supplementary material for: Emotion-induced loss aversion and striatal-amygdala coupling in low-anxious individuals
Source: Soc Cogn Affect Neurosci. 2015 Nov 19;11(4):569–79. doi: 10.1093/scan/nsv139 (PMC4814785; doi:10.1093/scan/nsv139)

***Supplementary Materials***

**Emotion-induced loss aversion and striatal-amygdala coupling in low anxious individuals**

**Authors:** Caroline J. Charpentier^1,3*^, Benedetto De Martino^2^, Alena L. Sim^1^, Tali Sharot^3^, Jonathan P. Roiser^1^

**Affiliations:** ^1^Institute of Cognitive Neuroscience, University College London, London WC1N 3AR, UK; ^2^Department of Psychology, University of Cambridge, Cambridge CB2 3EB, UK; ^3^Affective Brain Lab, Department of Experimental Psychology, University College London, London WC1H 0AP, UK.

***Corresponding author:** Caroline Charpentier, Institute of Cognitive Neuroscience, 17 Queen Square, London WC1N 3AR, UK. E-mail: [caroline.charpentier.11@ucl.ac.uk](mailto:caroline.charpentier.11@ucl.ac.uk). Telephone: +442076791138. Fax: +442078132835.

**Supplementary Methods**

*Stimuli*

Face stimuli consisted of pictures from the NimStim Face Stimulus Set (<http://www.macbrain.org/resources.htm>). A set of 40 identities was chosen (20 male faces and 20 female faces), and each face stimulus was presented depicting either a neutral, happy, or fearful expression, resulting in a set of 120 face stimuli (primes). For the object control condition, 20 pictures of light bulbs were selected. All stimuli were resized to a resolution of 200 (width) x 300 (height) pixels and were displayed on a black background using Cogent 2000 ([www.vislab.ucl.ac.uk/cogent.php](http://www.vislab.ucl.ac.uk/cogent.php)) running under Matlab.

*Practice memory task*

On the screening day, participants were initially presented with 20 trials of the memory task, with no gambles, thus ensuring that they were able to perform the task correctly and were familiar with the response buttons.

Specifically, on each trial participants memorized the positions of two or four faces or objects presented on the screen for 3 s, and, after a delay of 10 s, reported the previous location of one of the stimuli, displayed at the center of the screen. The stimuli displayed on each trial were chosen randomly among all pictures from the same emotion condition (all happy, all fearful, all neutral, or all objects) and (for faces) from the same gender (all males or all females). Difficulty and condition were randomized across trials.

*Gambling task*

The design of the gambling task to assess loss aversion was adapted from a previous study (Tom et al., 2007). On each trial participants were presented with a mixed gamble in which there was a 50% probability of winning the amount of money written in green (e.g. “WIN £12”) and a 50% probability of losing the amount written in red (e.g. “LOSE £8”). The left/right positions of the win and loss amounts were randomly assigned. Participants had 2 seconds to decide whether they wanted to take (accept) or pass on (reject) the gamble, and indicated their choice by a button press.

At the screening session, loss aversion was estimated in a training block of 49 trials (without the concurrent memory task) using a double staircase procedure to adjust the gambles for each participant. Potential wins and losses were varied parametrically as follows. The gamble expected value (EV = 0.5*win amount + 0.5*loss amount) was adjusted every 2 trials in order to reach the participant’s indifference point (the EV for which gambles were accepted half of the time on average). Each set of 2 trials contained one “high” EV gamble and one “low” EV gamble. The EV of accepted gambles was decreased by 0.5 while the EV of rejected gambles was increased by 0.5. Potential gains ranged between £6 and £24 and potential losses between £1 and £12. For each trial, the gain/loss pair was chosen randomly among all pairs with the same desired EV.

Participants finished the training session with 40 trials of the combined memory/loss aversion task, in which gambles were determined using a similar staircase procedure to the training gambling task above. From these data, we ensured that participants still scored above chance on the memory task despite interference from the gambling task, and that their choices on the gambles were consistent with their previously estimated indifference point.

*Incentive-compatible payment procedure*

Participants were endowed with an initial amount of £15, to which the average outcome of 10 randomly selected choices was added or removed. They were explained this payment procedure carefully before starting the task. Participants earned an average of £17.41 on the task (i.e. extra win of £2.41 on top of the initial £15 endowment), and payments ranged from £11 to £22.90. They also received £7 for their time on the screening session and £10 on the scanning session.

*Post-scanning tasks*

Immediately after scanning, participants completed a final block of 49 trials of the task, in which all the memory stimuli were objects. This block was added to control for the fact that during the main task objects were presented on only one-quarter of trials and might therefore be perceived as oddballs (relative to faces) and could potentially influence loss aversion purely on the basis of novelty. To control for this, the change in gambling propensity was additionally calculated using the data from the object-only block completed by participants immediately after scanning, instead of the object trials presented during the scan. The results were the same irrespective of which object condition was used for comparison in the non-emotional trials. The change in propensity to gamble between emotional and non-emotional trial significantly correlated with trait anxiety in both cases (object trials presented during the scan: r_(26)_=0.437, *P*=0.020; object trials from object-only block: r_(26)_=0.413, *P*=0.029). This argues against a possible oddball effect from the object trials.

Participants then rated each of the 120 faces used inside the scanner for emotional content and arousal, from very negative to very positive, and from not at all arousing to very arousing, respectively.

Finally they completed a debriefing questionnaire in order to determine whether they suspected the true purpose of the experiment. Only one participant indicated that he suspected the actual purpose, but also stated that this thought occurred to him while answering the debriefing questions and not while he was performing the task; therefore, his data were included in the analysis.

*Estimation of loss aversion parameter λ and choice consistency parameter µ*

A first Prospect Theory model (Eq. 1 and 2, see main text) was estimated with all trials included independently of emotion condition, in order to estimate loss aversion (λ) and choice consistency (µ) across all trials (Model 1). Both parameters were successfully estimated by our model in 28/30 participants. The remaining two participants’ choice behavior was very inconsistent (µ<0.8 and λ<0), resulting in poor model fits, and their data were excluded from the analyses. The mean loss aversion parameter (λ) was 1.56 (SD±0.92, range 0.63–5.44), significantly greater than 1 (one-sample t-test: t_(27)_=3.21, *P*=0.003).

In order to assess the impact of emotion on loss aversion and choice consistency, we estimated four further models:

- Model 2: λ and µ estimated separately for trials with emotional (happy and fearful faces) and non-emotional (neutral faces and objects) primes: 4-parameter model

- Model 3: λ and µ estimated separately for each of the four conditions (happy, fearful, neutral, and objects): 8-parameter model

- Model 4: λ estimated separately for emotion and no emotion trials; µ estimated separately for each of the four conditions: 6-parameter model

- Model 5: λ estimated separately for each of the four conditions; µ estimated separately for emotion and no emotion trials: 6-parameter model

To assess whether estimating λ and µ for emotional versus non-emotional contexts was more parsimonious than estimating them separately for each of the four conditions, the Bayesian Information Criteron (BIC) (Schwartz, 1978) was calculated for each model and each participant:

BIC = -2*LL + k*ln(N) (Eq. 3)

where k represents the number of parameters in the model, N the number of trials used to estimate the parameters, and LL the loglikehood of the model calculated using the estimated best fit parameters. Comparing BICs is similar to a loglikelihood ratio test with the addition that the number of parameters in the model is taken into account and, therefore, models with more parameters are penalized. Average BIC (natural log scale) across all participants for the two-condition model (emotional & non-emotional – Model 2) was 114.72 while average BICs for the other models including one or both parameters estimated separately for the four conditions (happy, fearful, neutral & object) were 130.22, 121.44 and 123.57 for Models 3, 4 and 5, respectively. The difference in BIC between Model 2 and the other models was at least 6.72, providing strong evidence in favor of the two-condition model (Kass and Raftery, 1995). Therefore the two-condition model was used preferentially in all analyses, other than to verify that the effects obtained were independent of valence or face processing *per se*.

*MRI data preprocessing*

The first four volumes of each functional session were discarded from the analyses to allow for T1 equilibration. A field map was then created for each functional session using the SPM FieldMap toolbox. Using this field map file for phase correction, images were realigned to the first functional volume of each session and unwarped using 7^th^ degree B-spline interpolation. Movement plots were checked to ensure that any scan-to-scan translations greater than one-half of a voxel (1.5 mm) or rotations greater than 1 degree did not cause artifacts in the corresponding scan(s). If artifacts were detected, the corrupted scans were removed and replaced by an average of the previous and following scans and the corrupted scan was added as a regressor of no interest in the design matrix. The anatomical scan was coregistered to the unwarped mean functional image. All images were then reoriented such that the anterior commissure lay at coordinates [x=0, y=0, z=0]. Functional images were spatially normalized to the standard Montreal Neurological Institute (MNI) EPI template using 7^th^ degree B-spline interpolation, and smoothed using a 4 mm^3^ full-width at half maximum (FWHM) Gaussian kernel. After defining and estimating contrasts, the resulting contrast images were smoothed again using a 7 mm FWHM kernel, such that the final images included in the second level models were smoothed by √(4^2^+7^2^) ≈ 8 mm.

**Supplementary Results**

**Emotional modulation of the propensity to accept gambles**

Increased loss aversion following emotional cues, as identified in low anxious individuals (see main text and Figure 2A) should be accompanied by a corresponding decrease in the propensity to gamble, as subjective utilities will be perceived as lower when losses loom larger. Consistent with this, there was a significant positive relationship between trait anxiety and the increase in the proportion of accepted gambles from non-emotional to emotional trials (r_(28)_=0.437, *P*=0.020).

**Choice consistency increases under emotion**

Choice consistency (μ) during gambling was significantly increased under emotional (fearful and happy stimuli together) trials relative to non-emotional (neutral and object stimuli together) trials (t_(27)_=2.24, *P*=0.033, Figure S1 and Table S1), suggesting that participants were more likely to make the same choice over repeated identical gambles, when primed with an emotional stimulus. However, the percentage change in choice consistency was not correlated with trait anxiety (r_(28)_=-0.31, *P*=0.12).

**Striatal loss aversion signals correlate negatively with trait anxiety**

In order to specifically identify regions in the brain where the parametric response to decreasing losses was greater than the parametric response to increasing gains, we explored the contrast [parametric response to losses > parametric response to gains] across the whole brain, independent of emotion condition.

We observed clusters surviving whole-brain correction for multiple comparisons in bilateral precuneus/occipital cortex and right ventral striatum (Table S2B and Figure S2A; the striatal region overlapped with that reported in the parametric analysis of expected value, see Figure 3A). In other words, responses in these regions tracked decreasing losses significantly more strongly than increasing gains (equivalent to the “neural loss aversion” signal identified by Tom et al., 2007). However, none of these activations was related to loss aversion behaviorally across participants, even when excluding an outlier with a very high loss aversion value (all r<0.25 and *P*>0.2).

Interestingly, biased striatal parametric responses to losses relative to gains (contrast estimate averaged across the cluster) were negatively correlated with trait anxiety (r_(28)_=-0.392, *P*=0.039, Figure S2B). Further analysis revealed that this effect was primarily driven by parametric response to losses: ventral striatum response to decreasing losses correlated significantly negatively with trait anxiety (r_(28)_=-0.478, *P*=0.01, Figure S2C), whereas the response to gains did not (r_(28)_=-0.016, *P*=0.94). These two correlations were significantly different (Steiger’s Z=1.98, *P*<0.05).

**Functional connectivity between ventral striatum and amygdala**

Functional connectivity across the entire fMRI time series (or “main effect” functional connectivity) was estimated from the physiological (striatal) regressor in our PPI model. The “main effect” connectivity between ventral striatum and amygdala was significantly positive (mean beta=0.111, SD=0.017, t_(27)_=6.60, *P*<0.0001), thus confirming pronounced coupling between ventral striatum and amygdala (Roy et al., 2009). There was no correlation between this “main effect” coupling and trait anxiety (r_(28)_=-0.14, *P*=0.48).

The emotional modulation of this functional connectivity between the ventral striatum and the amygdala (the PPI effect) was non-significant across all subjects (t_(27)_=0.76, *P*=0.46). In addition, an exploratory whole-brain analysis of the PPI effect (*P*<0.001 uncorrected, k>10 voxels) revealed no supra-threshold clusters where the connectivity with the ventral striatum seed region was modulated by emotion. Instead, this PPI effect in the amygdala varied across individuals according to their trait anxiety, in a way that predicted changes in loss aversion (see main text).

**References**

Kass RE, Raftery AE (1995) Bayes Factors. J Am Stat Assoc 90:773–795.

Roy AK, Shehzad Z, Margulies DS, Kelly a MC, Uddin LQ, Gotimer K, Biswal BB, Castellanos FX, Milham MP (2009) Functional connectivity of the human amygdala using resting state fMRI. Neuroimage 45:614–626.

Schwartz G (1978) Estimating the dimension of a model. Ann Stat 5:461–464.

Tom SM, Fox CR, Trepel C, Poldrack R a (2007) The neural basis of loss aversion in decision-making under risk. Science 315:515–518.

**Supplementary Figures**

**Figure S1. Choice consistency increases under emotion.** Choice consistency parameter (inverse temperature) μ was significantly higher on emotional relative to non-emotional trials. Two-tailed *P*-values: * *P*<0.05. Error bars represent SEM.

**
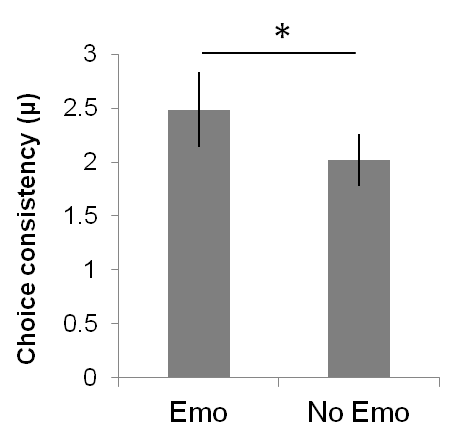
**

**Figure S2. Striatal loss aversion signals.** **A.** A cluster in the right ventral striatum showed a greater parametric response to decreasing losses than to gains. Activation is displayed at *P*<0.001 (uncorrected), but survived small volume correction (P_SVC_<0.05) in the anatomically defined striatal ROI (bilateral caudate + putamen). The color bar represents T-values and voxels are overlaid on the average anatomical scan from all 28 participants. **B.** Trait anxiety was negatively associated with the magnitude of this biased loss vs gain signal in the ventral striatum. **C.** Specifically, the parametric response to losses, but not to gains, was negatively correlated with trait anxiety: the less anxious the participant, the greater the response to decreasing losses, but not to increasing gains, in the ventral striatum.


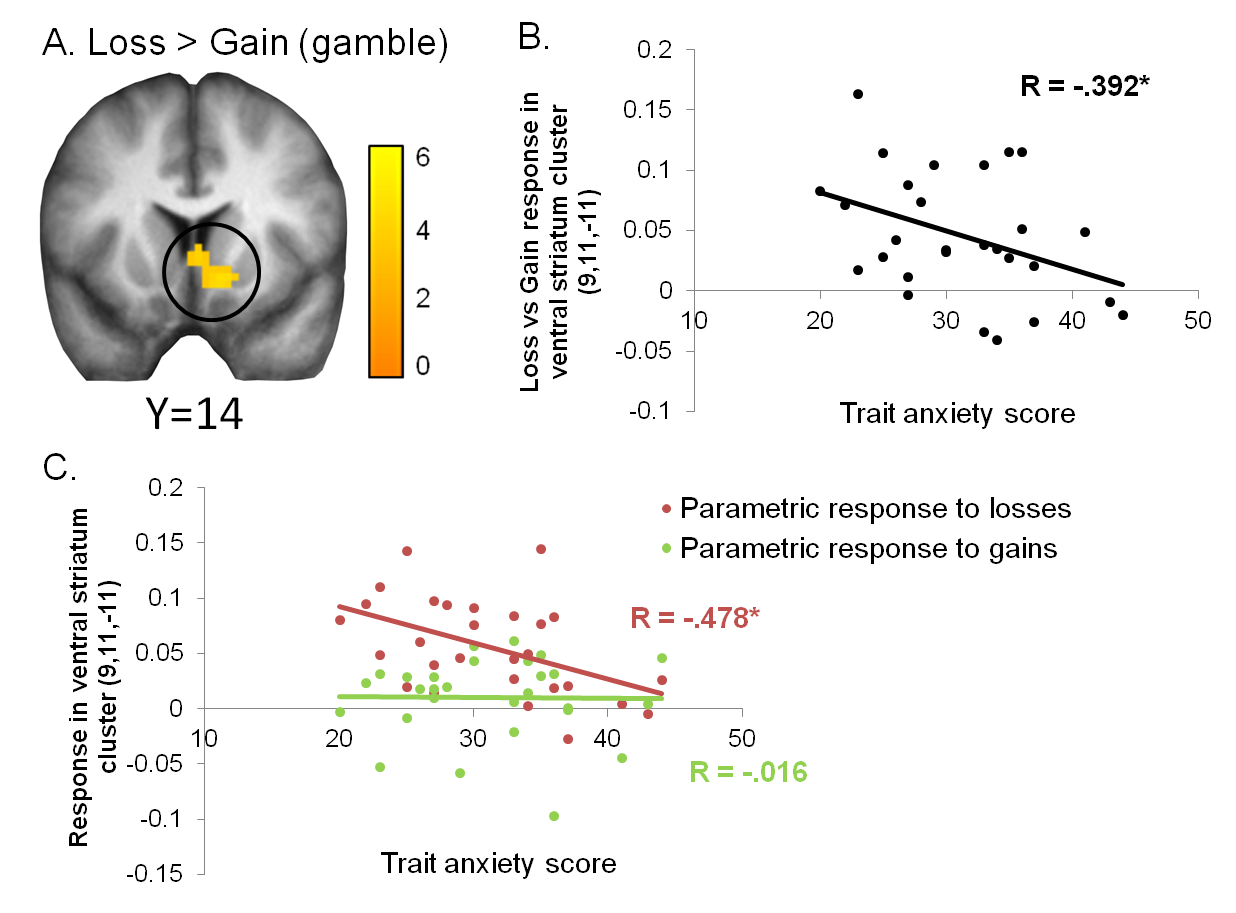


**Supplementary Tables**

**Table S1. Emotional modulation of task variables and interaction with trait anxiety.** For each condition, means, standard deviations, and ranges across participants are reported, for the following variables: probability to accept the gamble (P_accept_), loss aversion parameter (λ), reaction time to accept and reject the gamble in seconds (RT_accept_ and RT_reject_), number of missed gamble responses, working memory (WM) accuracy for the 2- and 4-stimulus conditions, number of missed memory responses, mean arousal and valence ratings (on a scale from 0 to 100). The main effect of condition and its interaction with trait anxiety were assessed and the corresponding statistics are reported in the last two columns. Apart from arousal and valence ratings, none of these variables were modulated by emotional stimuli. Only the emotional modulation of gamble acceptance and loss aversion (λ) varied according to trait anxiety. Averaged across all participants, the percentage change in loss aversion was not significantly different from zero (mean=1.01%, SD=7.8%, range=-18.08%-16.21%, one-sample t-test: t(27)=0.685, *P*=0.499).


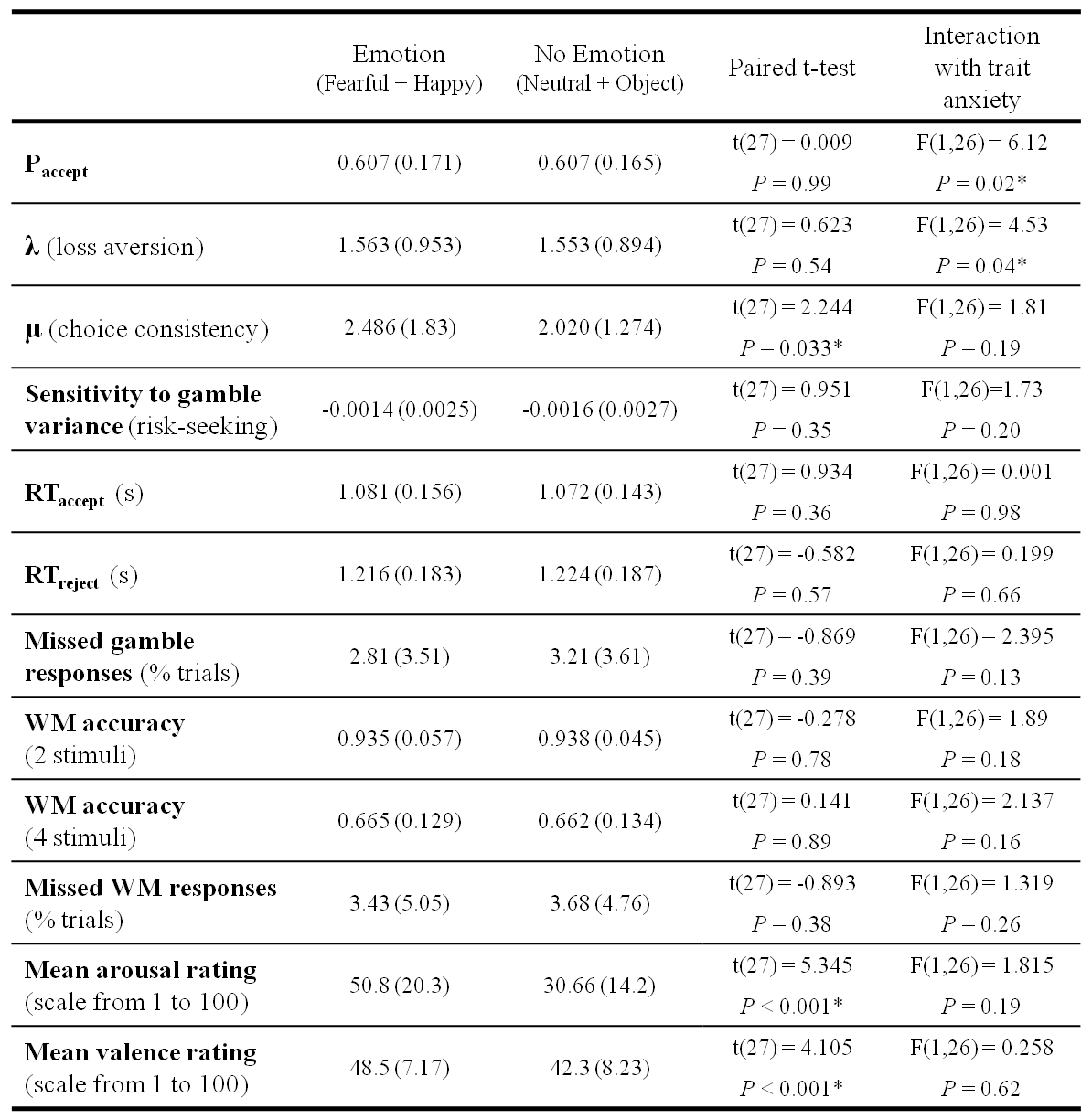


**Table S2. Brain regions exhibiting an expected value signal (A) and a neural loss aversion signal (B) at the time of gamble.** The analysis was initially thresholded at *P*<0.001 (uncorrected), cluster size ≥10. For completeness, clusters of at least 10 contiguous voxels that did not survive FWE correction are also reported. ACC – anterior cingulate cortex. OFC – orbitofrontal cortex.


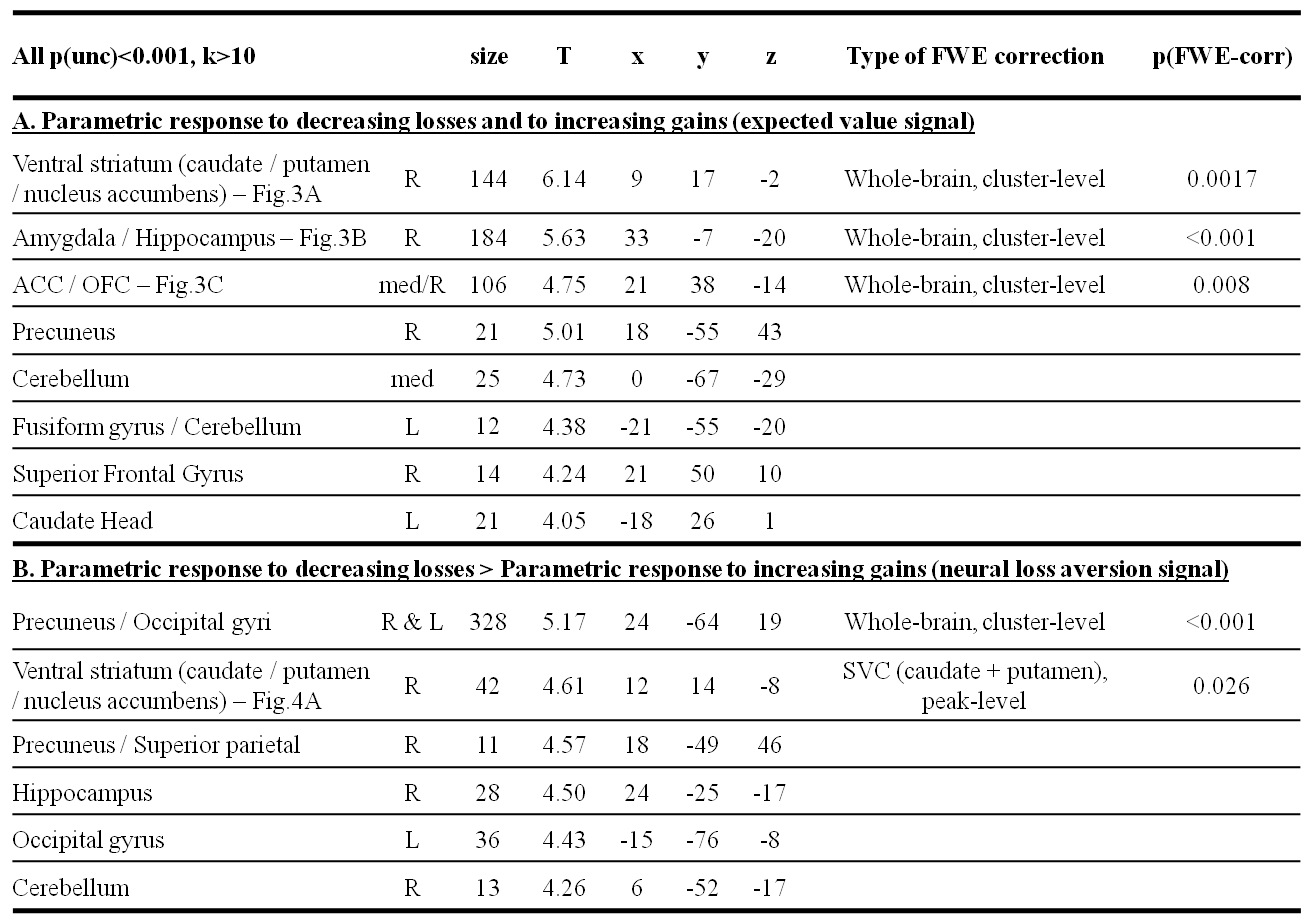


**Table S3. Brain regions showing response to emotional relative to non-emotional stimuli during presentation of the prime.** The analysis was initially thresholded at *P*<0.001 (uncorrected), cluster-size ≥10. To identify effects in the amygdala, a bilateral anatomical ROI was used to perform small volume correction without a cluster size criterion.


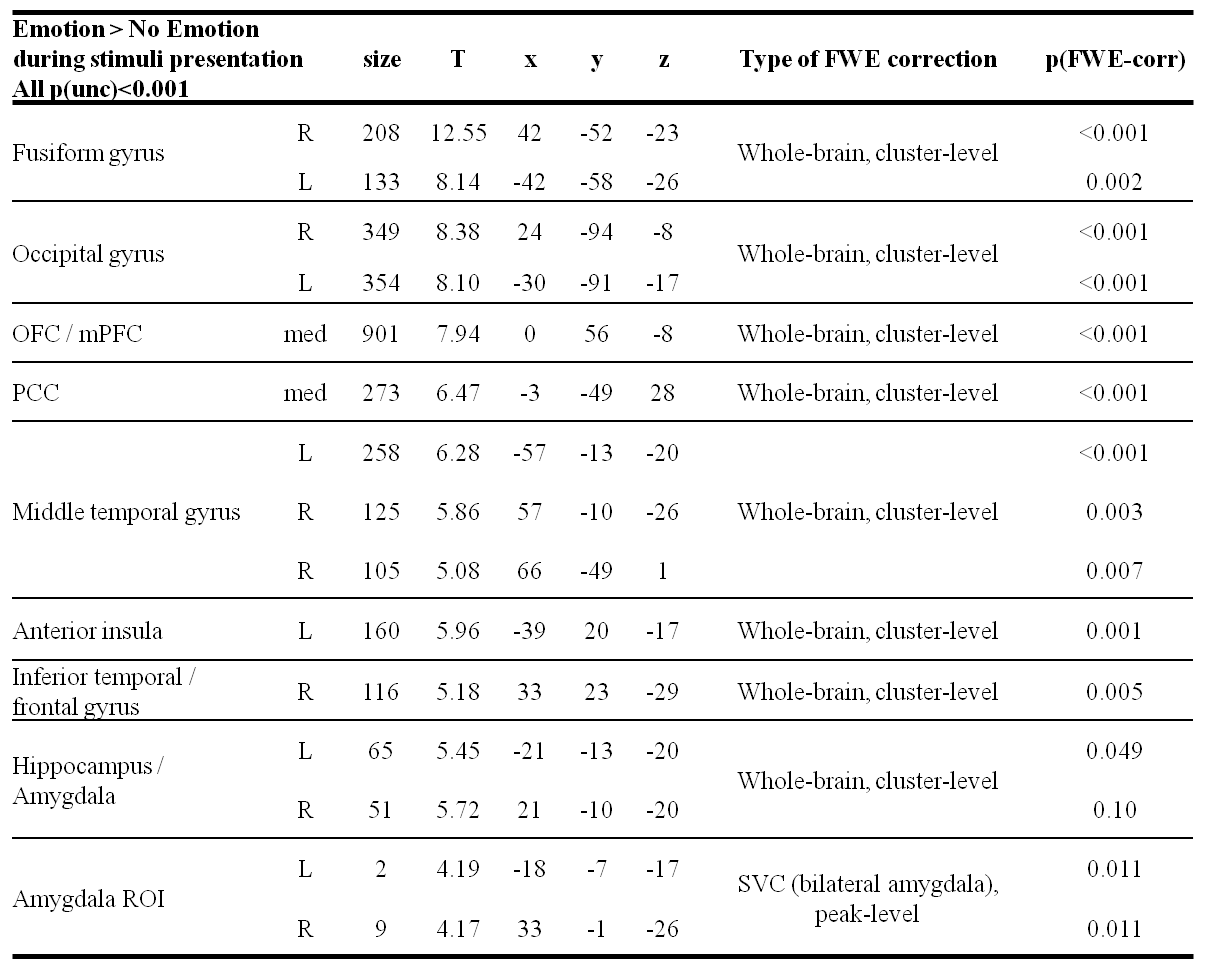

Supplement: Supplementary Data [file supp_nsv139_scan-15-297-File008.doc]
